# Supplementary material for: New Findings: Hindlimb Unloading Causes Nucleocytoplasmic Ca2+ Overload and DNA Damage in Skeletal Muscle
Source: Cells. 2023 Apr 3;12(7):1077. doi: 10.3390/cells12071077 (PMC10093444; doi:10.3390/cells12071077)
Supplement: Supplementary file 1 [file cells-12-01077-s001.zip › cells-2094888-SI.pdf]

Supplement Material

Figure S1. The negative control for Ca^{2+} fluorescence.

In order to exclude the influence of autofluorescence of biological tissues and other factors on the experimental results, we set up a negative control experiment.

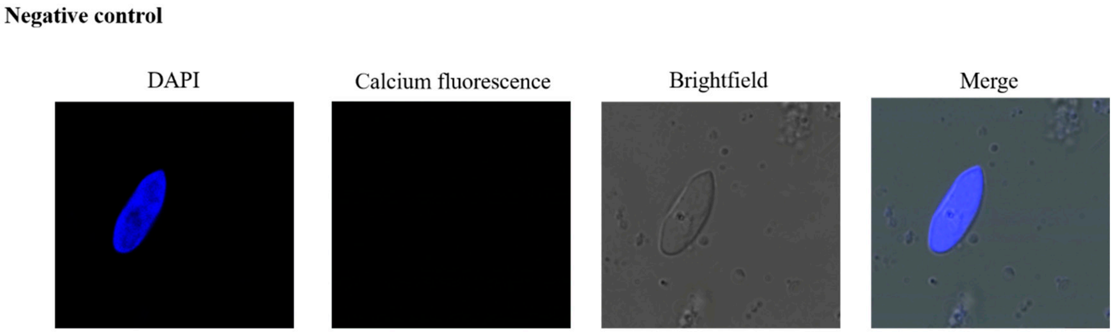

Figure S2. The sampling order and details of WB.

Note: Figure S2 is an umbrella term for the following figures, tables and text.
In our WB assay experiment, all the SDS-page gels contained 0.5% trichloroethanol (TCE), so the protein bands in such gel could be viewed under ultraviolet lamp. We selected one sample as the standard sample. By normalizing the density of the bands in standard sample among the different membranes, we could eliminate the display difference among the different membranes. Each lane in the gel contained 10 µg of protein. All the images were captured using a G:BOX XR5 Bioimaging system (Syngene, Cambridge, UK).

1. WB assay for RyR1; Ca^{2+}-ATPase2; CaMK II; PKA

We selected one sample in CON group as the standard sample in CON group, named CON-S. We uploaded all the CON samples into this gel:

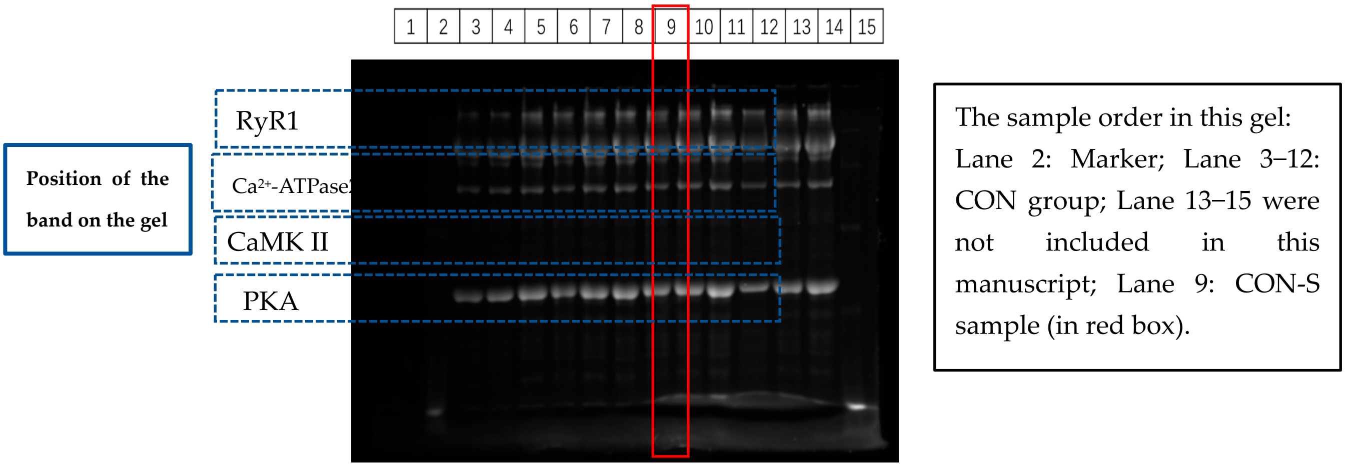

The picture of Gel 1

The target protein bands on the PVDF membrane derived from the above gel were as follow:

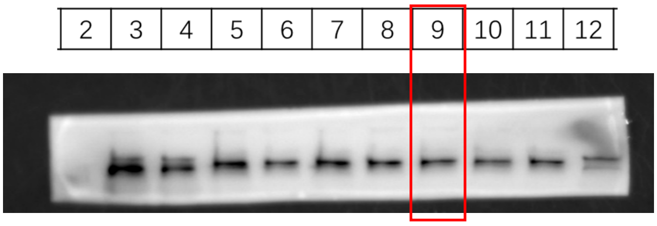	RyR1
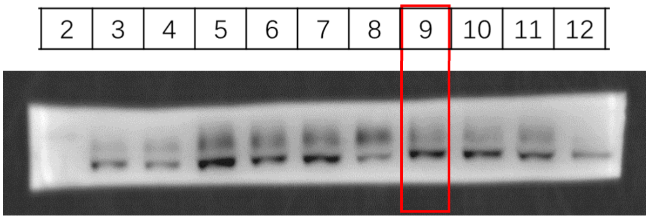	Ca^{2+}-ATPase2
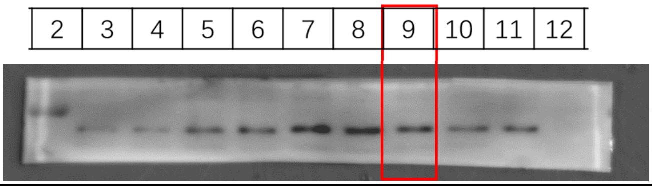	CaMK II
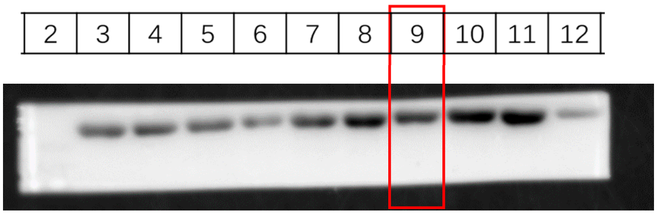	PKA

Similarly, we selected one sample in HLU group as the standard sample in HLU group, named HLU-S. We uploaded all the HLU samples into this gel:

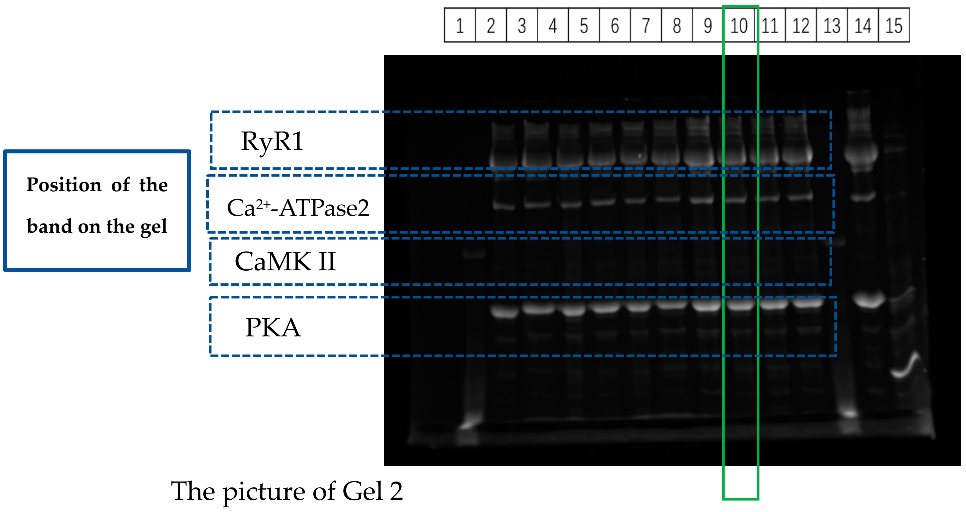

The sample order in this gel: Lane 2: Marker; Lane 3–12: HLU group; Lane 13–15 were not included in this manuscript; Lane 10: HLU-S sample (in green box)

The picture of Gel 2

The target protein bands on the PVDF membrane derived from the above gel were as follow:

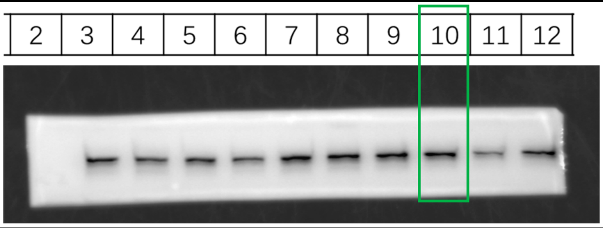	RyR1

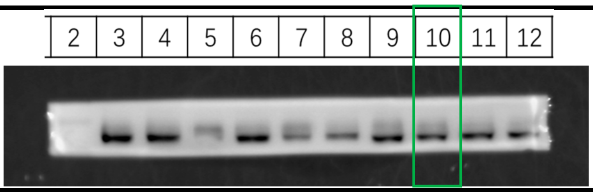

Ca^{2+}-ATPase2

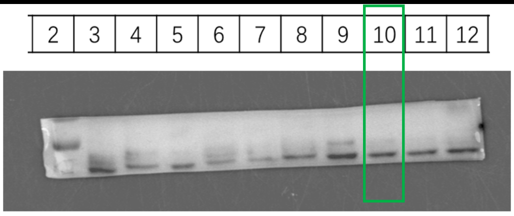

CaMK II

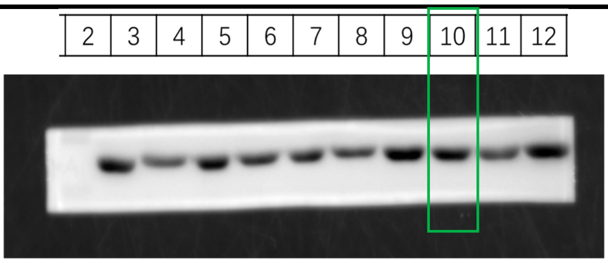

PKA

Eight samples (containing CON-S and HLU-S sample) from CON and HLU group were uploaded in one gel.

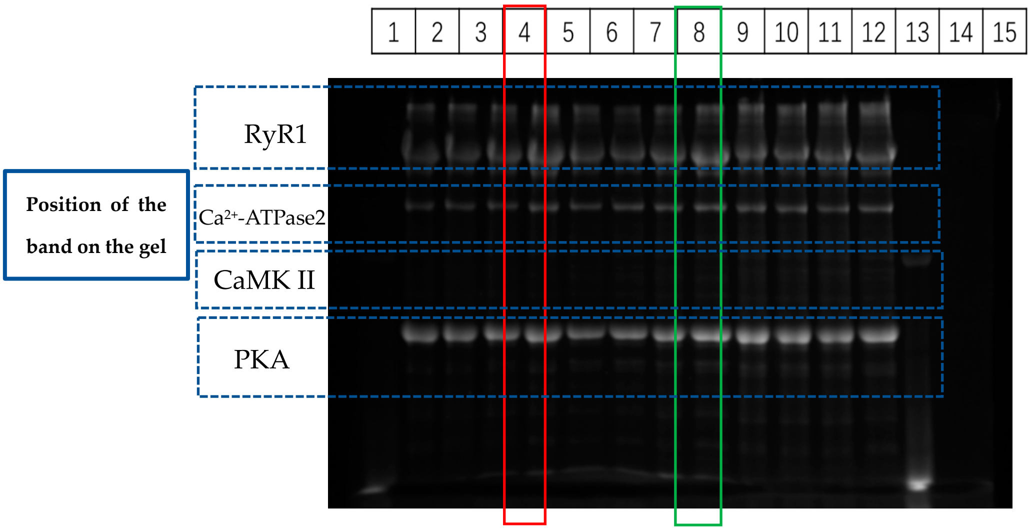

The sample order in this gel: Lane 1: Marker; Lane 2–5: CON group; Lane 6–9: HLU group; Lane 10–15 were not included in this manuscript; Lane 4: CON-S sample (in red box); Lane 8: HLU-S sample (in green box).

The picture of Gel 3

The target protein bands on the PVDF membrane derived from the above gel were as follow. Then we presented the band image of Lane 3–8 as the result image in the manuscript.

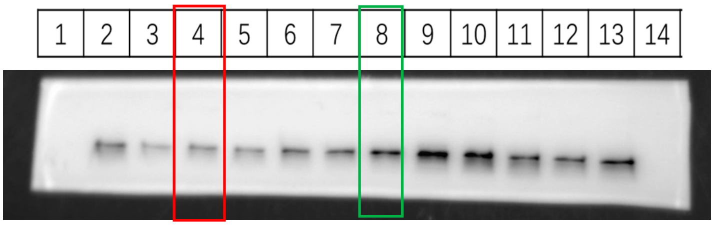

RyR1 (the band image in manuscript)

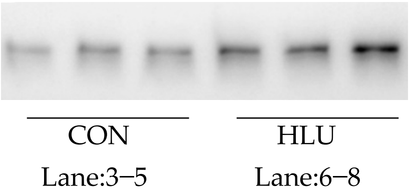

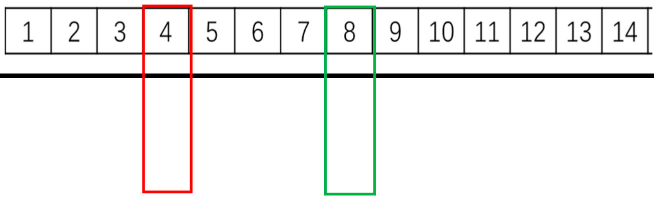

Ca^{2+}-ATPase2 (the band image in manuscript)

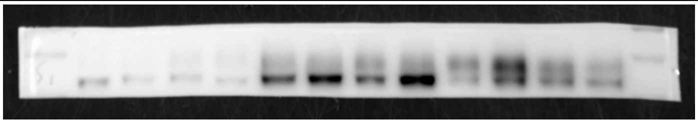	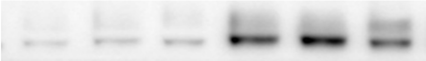
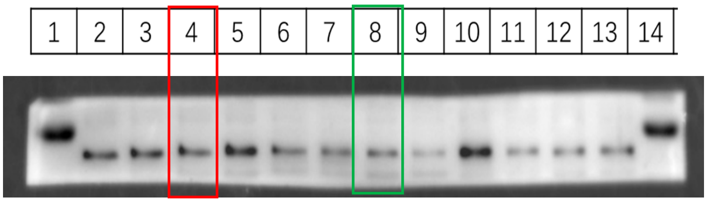	CaMK II (the band image in manuscript) 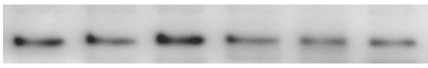
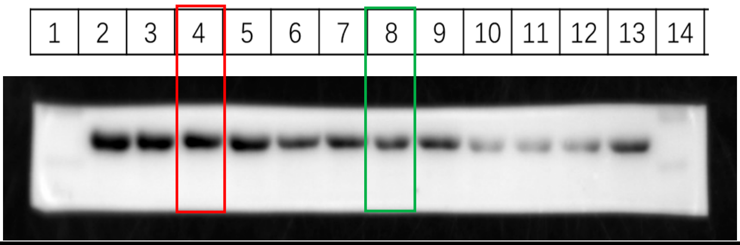	PKA (the band image in manuscript) 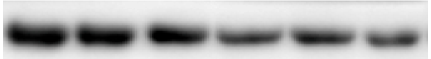

Combine with CON-S sample and HLU-S sample, all the target protein bands density was normalized by the band in CON-S sample.

2. WB assay for NCX3

Twelve samples (containing CON-S) from CON and HLU group were uploaded in one gel. The CON-S (in the red box) was used as the standard sample for this gel and all other lanes were normalized by this.

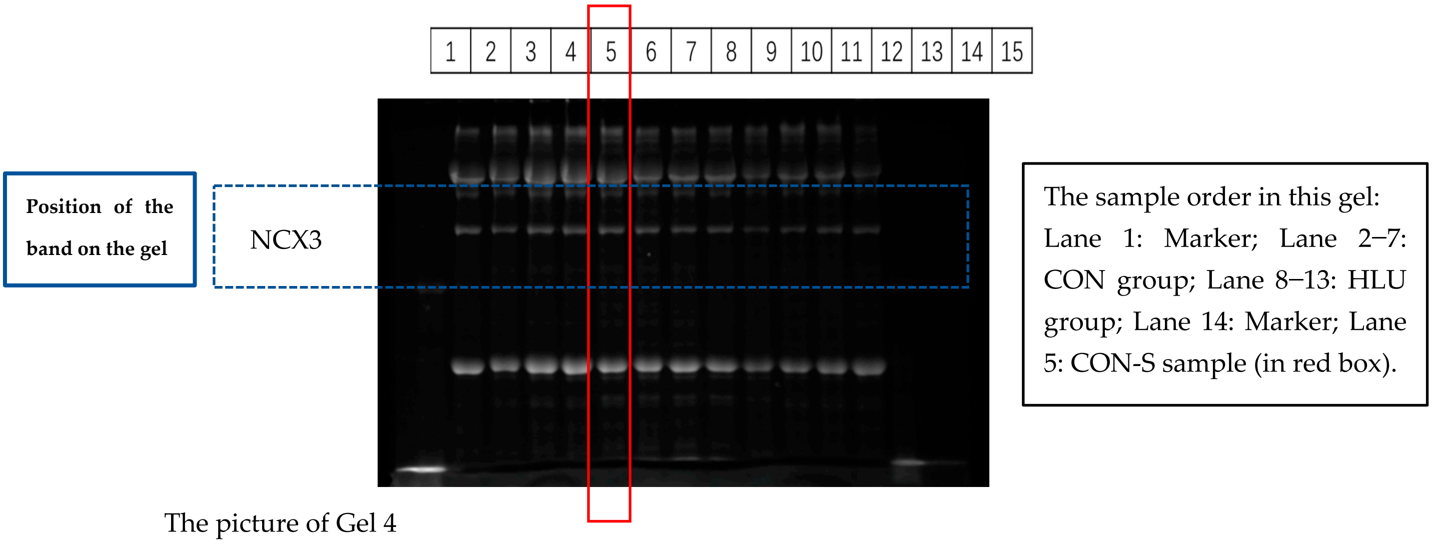

The picture of Gel 4

The target protein bands on the PVDF membrane derived from the above gel were as follow.

Then we presented the band image of Lane 5–10 as the result image in the manuscript.

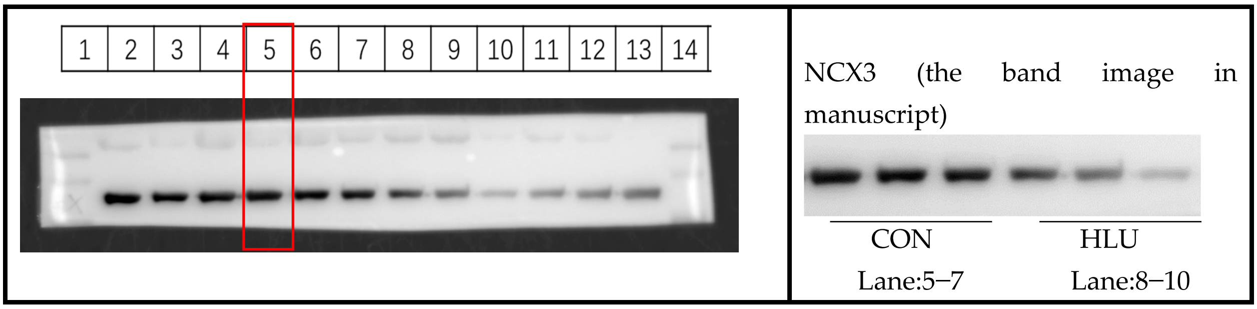

Based on the CON-S sample, all the target protein bands density was normalized by the band in CON-S sample.

3. WB assay for CD38

Twelve samples (containing CON-S) from CON and HLU group were uploaded in one gel. The CON-S (in the red box) was used as the standard sample for this gel and all other lanes were normalized by this.

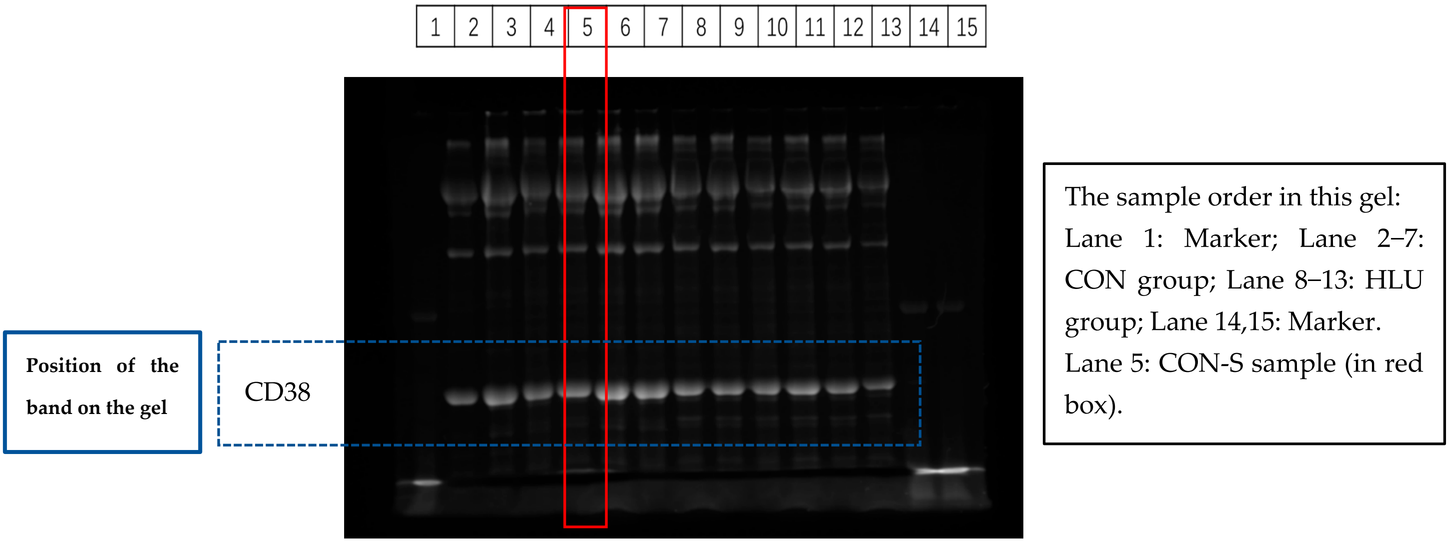

The picture of Gel 5

The target protein bands on the PVDF membrane derived from the above gel were as follow.

Then we presented the band image of Lane 5–10 as the result image in the manuscript.

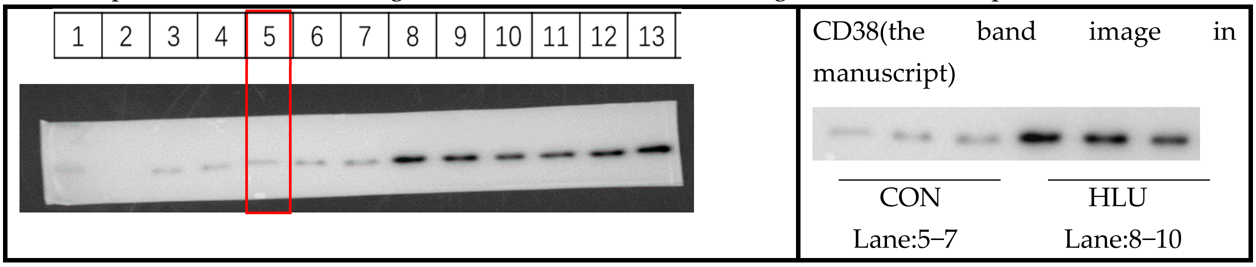

Based on the CON-S sample, all the target protein bands density was normalized by the band in CON-S sample.

4. WB assay for IP_3R1

Ten samples (containing CON-S) from CON and HLU group were uploaded in one gel. The CON-S (in the red box) was used as the standard sample for this gel and all other lanes were normalized by this.

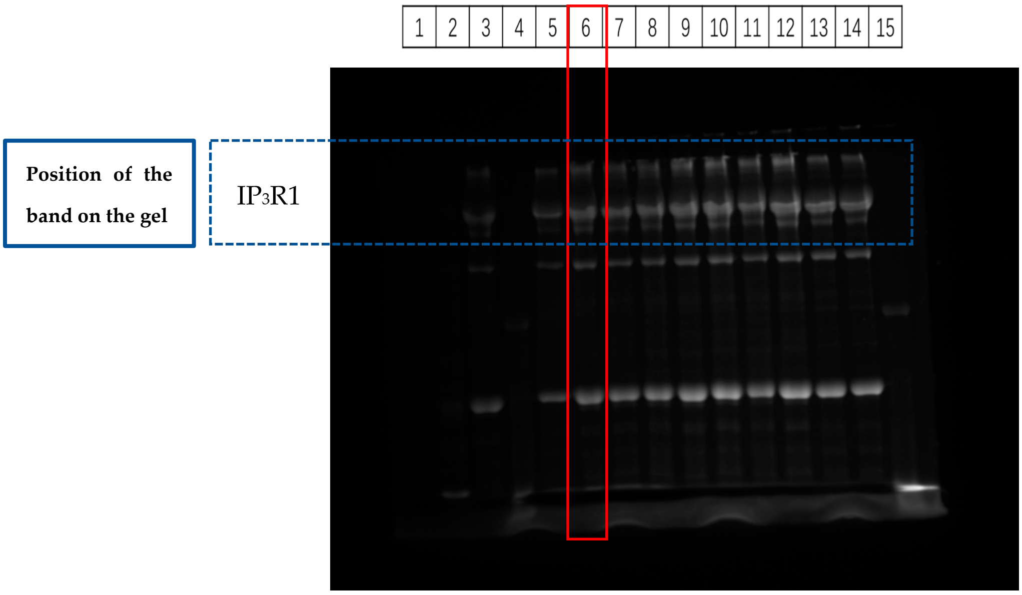

The sample order in this gel: Lane 1–4 were not included in this manuscript; Lane 5–8: CON group; Lane 9–14: HLU group; Lane 15: Marker; Lane 6: CON-S sample (in red box)

The picture of Gel 6

The target protein bands on the PVDF membrane derived from the above gel were as follow.
Then we presented the band image of Lane6–11 as the result image in the manuscript.

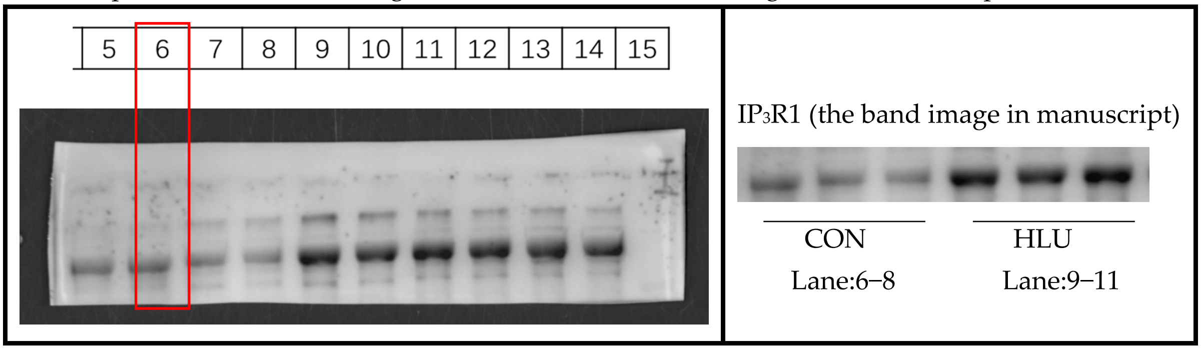

Based on the CON-S sample, all the target protein bands density was normalized by the band in CON-S sample.

Six samples (containing CON-S) from CON and HLU group were uploaded in one gel. The CON-S (in the red box) was used as the standard sample for this gel and all other lanes were normalized by this.

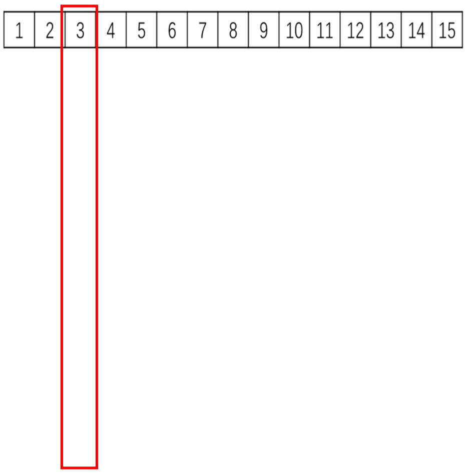

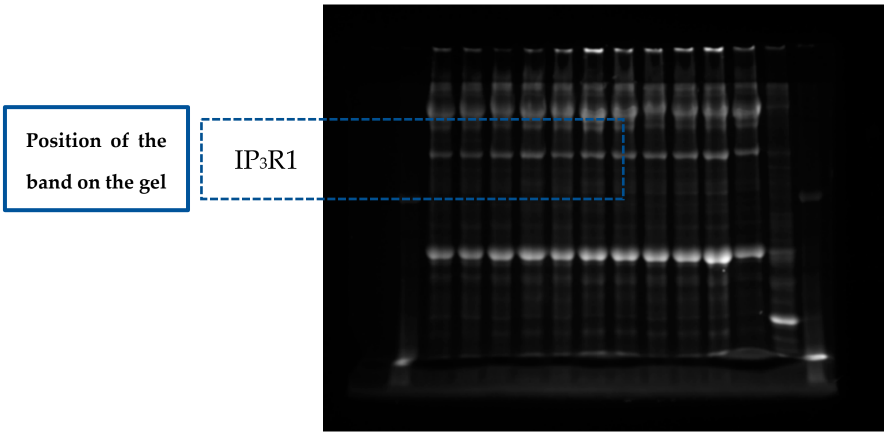

The sample order in this gel: Lane 2: Marker; Lane 3–5: CON group; Lane 6–8: HLU group; Lane 9–15 were not included in this manuscript; Lane 3: CON-S sample (in red box).

The picture of Gel 7

The target protein bands on the PVDF membrane derived from the above gel were as follow.

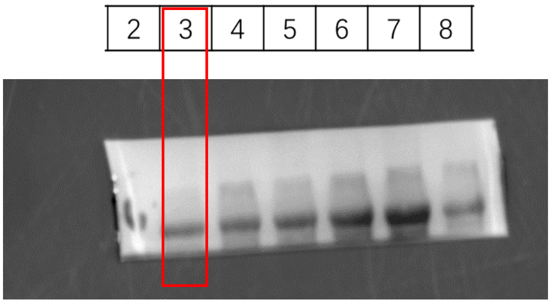

IP3R1

Based on the CON-S sample, all the target protein bands density was normalized by the band in CON-S sample.

Figure S3. Dystrophin and PCM1 immunofluorescence double staining.

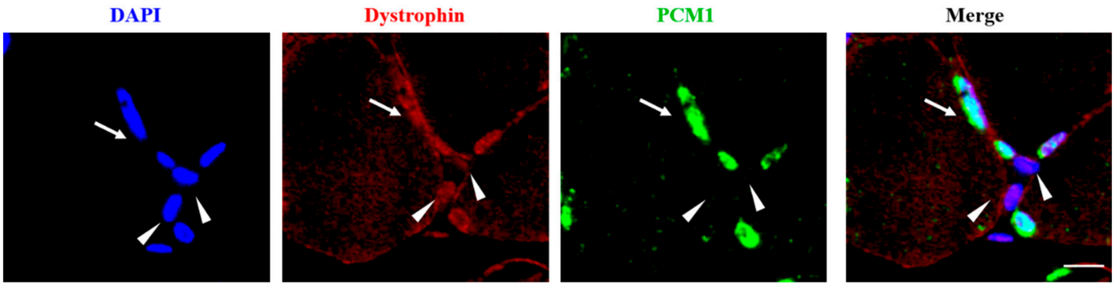

Figure S3. Dystrophin and PCM1 immunofluorescence double staining on soleus muscle sectioning slice. The dystrophin were red and the PCM1 were green after immunofluorescence staining. In Merge, the nuclei located inside the plasma membrane were co-localised with PCM1 (myocyte nuclei, indicated by an arrow), and the nuclei located outside the plasma membrane were not co-localised with PCM1 (non-myocyte nuclei, indicated by an arrowhead). Scale bar, 10 μm.

Figure S4. The negative control for Immunofluorescence.

In order to exclude the influence of autofluorescence of biological tissues and other factors on the experimental results, we set up a negative control experiment.

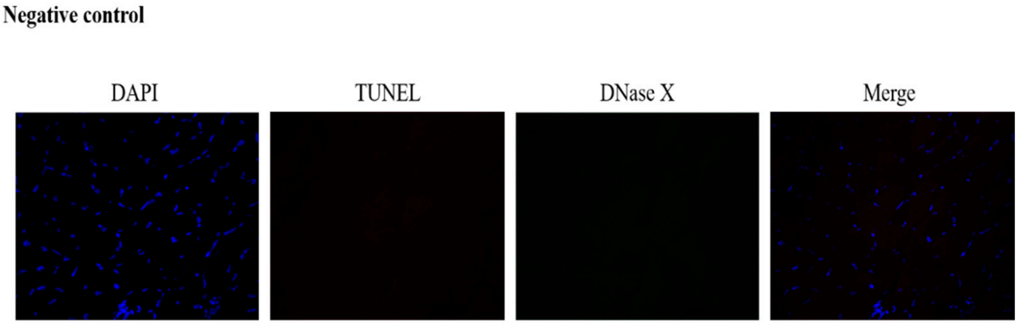

Figure S5. The phosphorylation levels of CaMK II in the nucleus.

We have done a supplementary experiment to detect the phosphorylation level of CaMK II in the nucleus. As shown in Fig. S5 in this file, the results showed that the phosphorylation level of CaMK II in the nucleus significantly increased by 132% in the HLU group compared to the CON group. It indicated that the nuclear Ca^{2+} level in the HLU group was higher than that in the CON group.

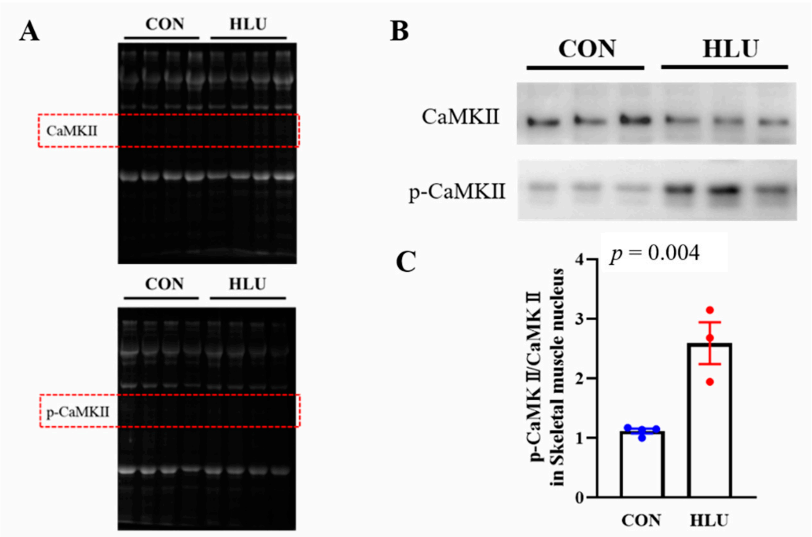

Figure S5. Phosphorylation levels of CaMK II in nucleus. (A) Gels of CaMK II and p-CaMK II. (B) The bands of CaMK II and p-CaMK II. (C) Changes in the phosphorylation levels of CaMK II (p-CaMK II / CaMK II) in two groups. n = 3–4. Data were analyzed by *t*-test. Data were shown as Mean ± SEM and considered statistically significant at $p < 0.05$.
